# Human thymus medullary epithelial cells promote regulatory T-cell generation by stimulating interleukin-2 production via ICOS ligand

**DOI:** 10.1038/cddis.2014.377

**Published:** 2014-09-11

**Authors:** D Nazzal, A Gradolatto, F Truffault, J Bismuth, S Berrih-Aknin

**Affiliations:** 1 INSERM U974, Paris, France; 2 CNRS FRE3617, Paris, France; 3 Sorbonne Universités, UPMC Univ Paris 06, UM76, Paris, France; 4 AIM, Institut de Myologie, Paris, France

## Abstract

Natural thymic T regulatory (tTreg) cells maintain tolerance to self-antigen. These cells are generated in the thymus, but how this generation occurs is still controversial. Furthermore, the contribution of thymus epithelial cells to this process is still unclear, especially in humans. Using an exceptional panel of human thymic samples, we demonstrated that medullary thymus epithelial cells (mTECs) promote the generation of tTreg cells and favor their function. These effects were mediated through soluble factors and were mTEC specific since other cell types had no such effect. By evaluating the effects of mTECs on the absolute number of Treg cells and their state of proliferation or cell death, we conclude that mTECs promote the proliferation of newly generated CD25+ cells from CD4+CD25− cells and protect Treg cells from cell death. This observation implicates Bcl-2 and mitochondrial membrane potential changes, indicating that the intrinsic cell death pathway is involved in Treg protection by mTECs. Interestingly, when the mTECs were cultured directly with purified Treg cells, they were able to promote their phenotype but not their expansion, suggesting that CD4+CD25− cells have a role in the expansion process. To explore the mechanisms involved, several neutralizing antibodies were tested. The effects of mTECs on Treg cells were essentially due to interleukin (IL)-2 overproduction by thymus CD4+ T cells. We then searched for a soluble factor produced by mTECs able to increase IL-2 production by CD4+ cells and could identify the inducible T-cell costimulator ligand (ICOSL). Our data strongly suggest a « *ménage à trois* »: mTEC cells (via ICOSL) induce overproduction of IL-2 by CD25− T cells leading to the expansion of tTreg cells. Altogether, these results demonstrate for the first time a role of mTECs in promoting Treg cell expansion in the human thymus and implicate IL-2 and ICOSL in this process.

The thymus is the primary lymphoid organ of T-lymphocyte maturation. Immature thymocytes undergo positive selection in the thymic cortex, followed by negative selection in the thymic medulla. T-cell development necessitates constant input from stromal thymus cells via cell–cell interactions and soluble factors. Disturbances of one or the other processes can favor immune dysregulation.^1^ Developing thymocytes receive a wide array of signals from thymic epithelial cells (TECs) for selection, survival, expansion, and differentiation, which can result either in cell death or in differentiated self-tolerating T cells.^2, 3^ The importance of TECs for the development of self-tolerant T cells is highlighted by autoimmunity and immunodeficiencies that can occur during abnormal development.^1, 4^

T regulatory (Treg) CD4+CD25+ cells prevent the activation of auto-reactive T cells and have a key role in the induction of peripheral tolerance *in vivo.*^5^ Treg cells can be produced in the thymus or in the periphery. The suppressive function of Treg cells depends on the expression of the forkhead box P3 (FoxP3) transcription factor. Thymus Treg (tTreg^6^) cells constitutively express the FoxP3 marker and have an essential role in maintaining tolerance to self-antigens.^7^ In contrast, peripheral Treg (pTreg^6^) cells are conventional T cells (Tconv, CD4+CD25−) that express FoxP3 and CD25 transiently upon activation.^8^ tTreg cells have an important role in autoimmune diseases^5, 9^ as thymectomy of animal neonates leads to the development of autoimmune pathologies^10^ and their implication in different autoimmune diseases has been thoroughly investigated.^8^ Considering that tTreg cells are produced within the thymus and that studies investigating their role typically have used Treg cells isolated from whole blood (that is, predominantly pTreg cells) or animal models,^8, 11, 12, 13^ studies of human tTreg cells are rare.

tTreg cells are generated in the thymus medulla, mainly from CD4 single positive precursors.^11, 14^ Some studies suggest that tTreg cells originate from CD4+CD25+ FoxP3− cells that start to express FoxP3 later,^15^ a timing mechanism that would explain why tTreg cells are not detected in the thymus of newborn mice until 4 days after birth.^10^ Different subsets of stromal cells have been shown to favor tTreg cell development. In humans, dendritic cells (DCs) promote tTreg cell differentiation after activation by the thymus stromal lymphopoietin (TSLP) produced by medullary TECs (mTECs) from Hassall's corpuscles.^16, 17^ Plasmacytoid DCs are also able to promote tTreg cell differentiation under CD40L and interleukin (IL)-3 activation.^18^ In a mouse model where major histocompatibility complex (MHC) class II was only expressed by cortical TECs (cTECs), Liston *et al.*^12^ showed that these cells can be sufficient for tTreg cell differentiation. mTECs have a role in the selection of tTreg cells via T-cell receptor (TCR) activation and MHC class II presentation.^19^ TCR activation in tTreg cells is thought to take place in a small number of developmental niches within the thymus, with each niche of developing T cells corresponding to an antigen with a specific tolerance.^11^ Whereas IL-2 is important for tTreg cell development,^10, 20, 21^ other cytokines that possess the same gamma chain, such as IL-7 and IL-15, have a complementary role and can induce FoxP3 expression.^21, 22^ Although different cell types can have a role in tTreg cell development, mTECs have been consistently suggested to be important for tTreg cell differentiation, even though direct evidence for their role has not yet been elucidated.^10, 23^

The present study shows for the first time that thymus CD4+ or CD4+CD25− T cells cultured in the presence of human mTECs are more likely to display Treg cell phenotypes and functions. This effect is mediated through soluble factors and is due to the proliferation and protection of newly differentiated CD4+CD25+ T cells, which involves interactions between mTECs and CD4+ T cells and requires inducible costimulator on activated T-cell ligand (ICOSL) and IL-2.

## Results

### FoxP3+ cells are localized close to mTECs in the medullary area of the human thymus

**Figure 1:**
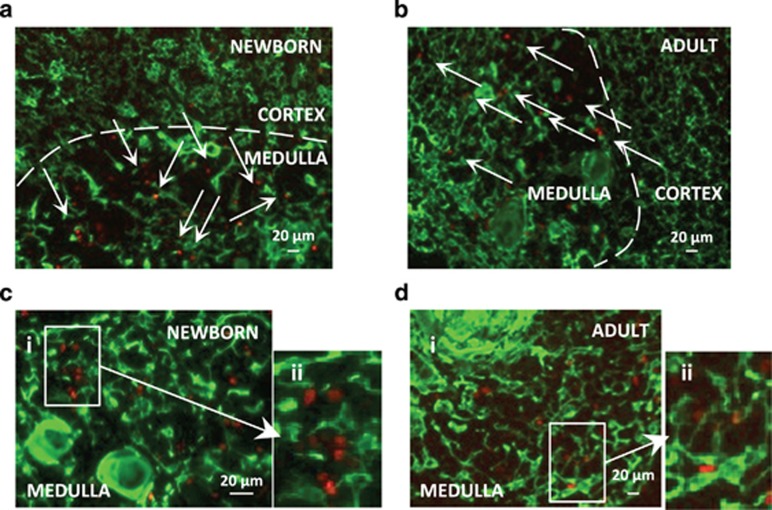
FoxP3+ cells are localized close to TECs in the medullary area of the human thymus. FoxP3+ cells were stained in red and TECs (keratin-positive) were stained in green on 7 *μ*m human thymus frozen sections. (**a** and **b**) In both the newborn (**a**; female 6 months old; magnification × 100) and adult (**b**; female 22 years old; magnification × 100) thymus, FoxP3+ cells (arrows) are mainly localized in the medulla. (**c** and **d**) FoxP3+ cells are present next to TEC in the medullary area of the newborn (**c**; female 6 months old; magnification × 200) and adult thymus (**d**; female 22 years old; magnification × 100). Panels **c**ii and **d**ii are high-magnification (twofold) views of **c**i and **d**i, respectively

FoxP3+ cells are present in the mouse thymus medulla and appear after 4 days of life.^10^ Although few studies have been conducted in humans, tTreg cells can be detected in the neonatal thymus.^24^ Here, we identified FoxP3+ cells in newborn and adult human samples and showed that the majority of these cells are localized in medullary areas (Figures 1a and b). Costaining shows that FoxP3+ cells are close to mTECs (Figures 1ci, ii and di, ii).

Since mTECs have a major role in T-cell development and are geographically close to tTreg cells, we investigated whether mTECs could affect Treg cell phenotype and function.

### mTECs regulate Treg cell phenotype

To determine the effects that mTECs exert on Treg cells, we used a coculture of well-established human mTECs (previously described in Cufi *et al.*^25^ Supplementary Figure S1) with purified human thymic CD4+ cells. The expression of CD25 and FoxP3 was evaluated by gating the live CD4+ cells as described in Supplementary Figure S2.

**Figure 2:**
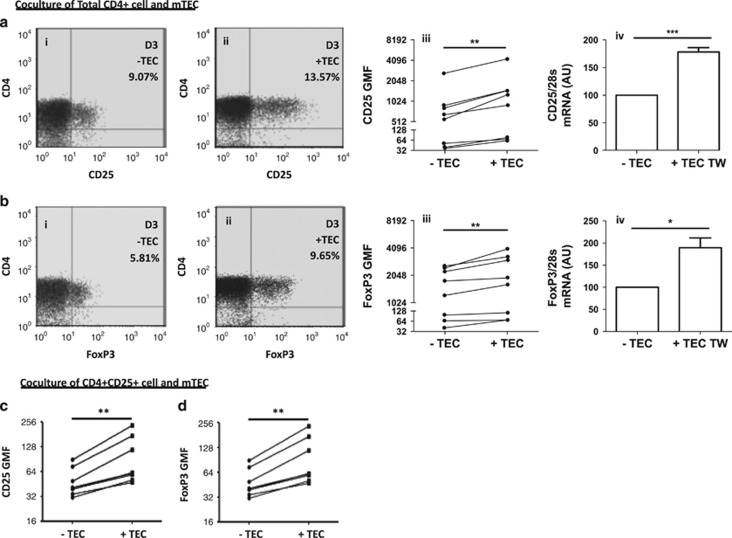
mTECs protect and enhance Treg cell phenotype. Purified CD4+ (**a** and **b**) or CD4+CD25+ T cells (**c** and **d**) were cultured alone or with mTECs for 3 days. (**a** and **b**) The percentages of CD25+ and FoxP3+ cells (**a**i, ii and **b**i, ii) and their GMF (**a**iii and **b**iii, log_2_ axis, *n*=10, mean±S.E.M.) were then evaluated. Expression of CD25 (**a**iv) and FoxP3 (**b**iv) was estimated via qPCR analyses (*n*=5, mean±S.E.M.). (**c** and **d**) Both CD25 (*n*=7) and FoxP3 (*n*=5) GMFs were assessed using flow cytometric analyses (log_2_ axis). Statistical analyses were conducted using non-parametric tests; *P<0.05, **P<0.005, ***P<0.001

Coculture of CD4+ T cells with mTECs enhances the percentage of CD25+ (Figures 2ai and ii) and FoxP3+ cells (Figures 2bi and ii) after 3 days of culture. The majority of CD25+ cells were also FoxP3+ in both the conditions (with or without mTECs) (Supplementary Figure S4). We noted a similar observation for the geometric mean fluorescence (GMF) of CD25 (Figure 2aiii) and FoxP3 (Figure 2aiii), which both showed higher expression in the presence of mTECs in all of the experiments (*n*=10). These results suggest that mTECs favor Treg cell production. Moreover, quantitative real-time PCR (qPCR) analysis revealed increased mRNA levels for both CD25 (Figure 2aiv) and FoxP3 (Figure 2biv) expression, suggesting transcriptional regulation. However, comparison with the D0 value (Supplementary Figure S3) suggests that the percentage of CD25 is slightly enhanced in CD4–mTECs cocultures (Supplementary Figure S3a), whereas the percentage of FoxP3 is only preserved (Supplementary Figure S3b).

We then asked whether mTECS would have a similar effect on purified CD4+CD25+ cells. A coculture of CD4+CD25+ cells with mTECs favored higher GMF expressions of CD25+ (Figure 2c) and FoxP3+ (Figure 2d) cells, suggesting that mTECs have a direct effect on Treg cells.

These observations suggest that mTEC and Treg cell interactions are important to maintain the FoxP3 expression and protect the Treg cell phenotype.

### mTECs specifically favor the Treg cell phenotype

**Figure 3:**
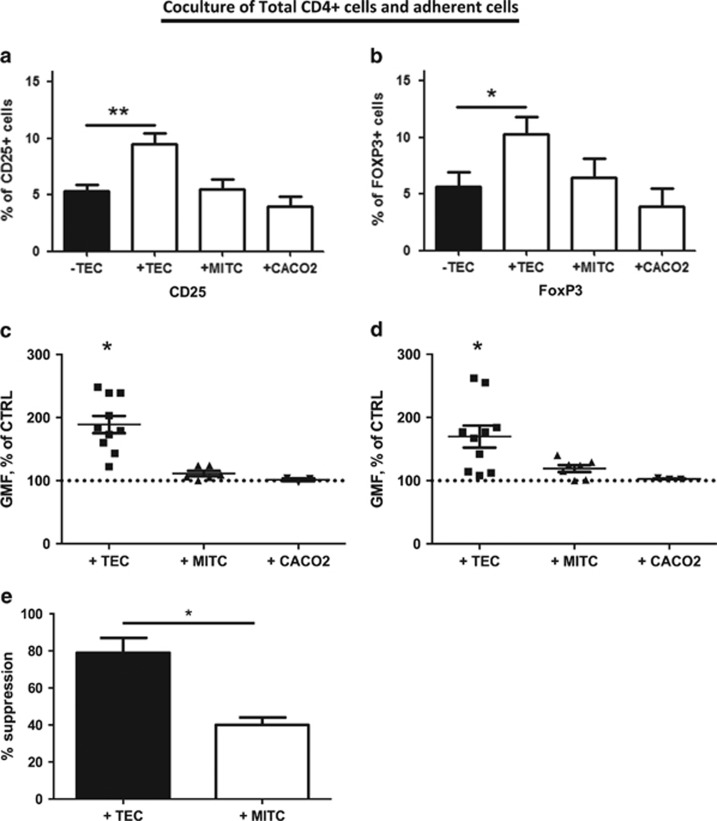
The protection of Treg cell phenotype is mTEC specific. Purified CD4+ T cells were cultured alone or with mTECs, MITCs, or CACO2 for 3 days. Panels **a** and **b** show the percentage of CD25+ and FoxP3+ cells, respectively (*n*=6–13, mean±S.E.M.). Panels **c** and **d** show the GMF of CD25+ or FoxP3+ cells under the different culture conditions, respectively (*n*=3–10, mean±S.E.M.; percentage normalized to control conditions without mTECs). The dotted line represents the 100% reference for each marker. Statistical analyses were conducted using non-parametric tests; *P<0.05, **P<0.005. (**e**) The suppressive activity of CD4+CD25+ cells following 3 days of culture with mTECs or MITCs was evaluated by tritiated thymidine incorporation, as previously described in the literature.^45^ The suppressive capacity of Treg cells was normalized as the percentage of proliferative response of Tconv cells alone (*n*=3, mean±S.E.M.). Statistical analysis were conducted using a *t*-test

To investigate whether the effects of mTECs are specific, we tested cocultures of total CD4+ T cells with other adherent cell lines. When CD4+ cells were cocultured with myoid immortalized thymic cells (MITCs) or CACO2 cells, no favoring of the Treg cell phenotype was observed either in the percentage of positive cells (Figures 3a and b) or in their GMF values (Figures 3c and d). These findings were associated with Treg cell functionality, as CD25+ cells sorted from mTEC cocultures were still able to suppress their CD25− counterparts (80% suppression), whereas cells sorted from MITC coculture did not (40% suppression; Figure 3e).

These results demonstrate that mTECs, but not MITCs or CACO2 cells, specifically favor Treg cell phenotype and function.

### Direct contact between T cells and mTECs is not required for the maintenance of the Treg cell phenotype

**Figure 4:**
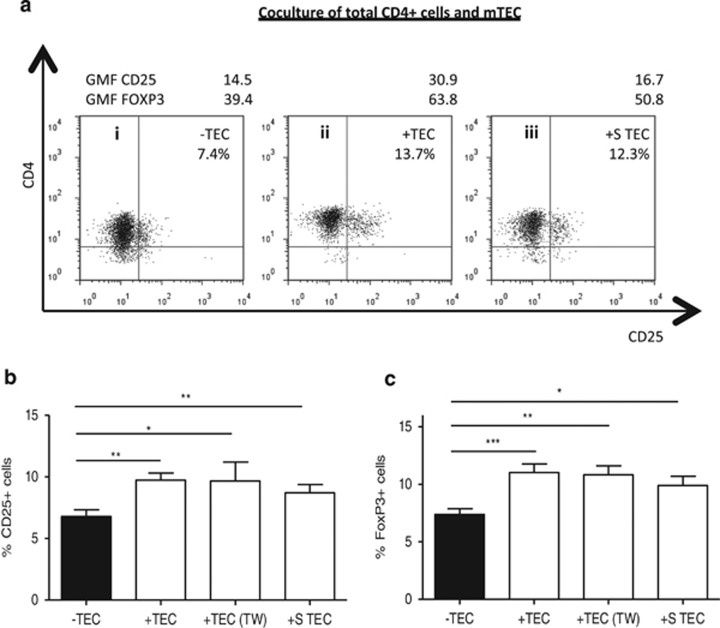
Direct contact between CD4+ cells and mTECs is not required for the maintenance of the Treg cell phenotype. Purified CD4 cells were cultured alone or with mTECs for 3 days. In some experiments, CD4 T cells were separated from mTECs by TWs. (**a**) Representative analysis of the percentages and GMFs of CD25+ and FoxP3+ cells, from CD4+ cells cultured alone (i), indirect contact with mTECs (ii), or with S-TEC (iii). Panels **b** and **c** show a summary of independent experiments with CD4+ cells cultured alone, with mTECs (*n*=10) or with mTECs separated with TW (*n*=6) or with S-TEC (*n*=4). Results are expressed as mean±S.E.M. Statistical analyses made all comparisons with the condition without mTECs, using the non-parametric paired *t*-test; *P<0.05, **P<0.005, ***P<0.001

To investigate whether the effects of mTECs on CD4+ cells required direct cell–cell contact, we performed cocultures using transwells (TWs) or cultures with supernatants issued from previous mTEC cultures (S-TEC). When CD4+ cells were cultured with S-TEC or in TWs, we still observed an increase in CD25 and FoxP3 percentages and GMFs (Figures 4a–c), suggesting that the effects of mTECs are mainly mediated through soluble factors.

### mTECs protect newly generated CD25+ cells from cell death and induce preferential proliferation of CD25+ cells

The effects of mTECs on the Treg cell phenotype (Figure 2) suggest at least four non-mutually exclusive possibilities: higher differentiation of CD25− into CD25+ cells, less death of CD25+ cells, higher proliferation of CD25+ cells, or preferential death of CD25− cells. To test these hypotheses, we performed cocultures of mTECs with highly purified and carboxyfluorescein diacetate succinimidyl ester-stained (CFSE) CD25− Tconv cells (up to 99.3% viable cells at D0; Supplementary Figure S5a).

**Figure 5:**
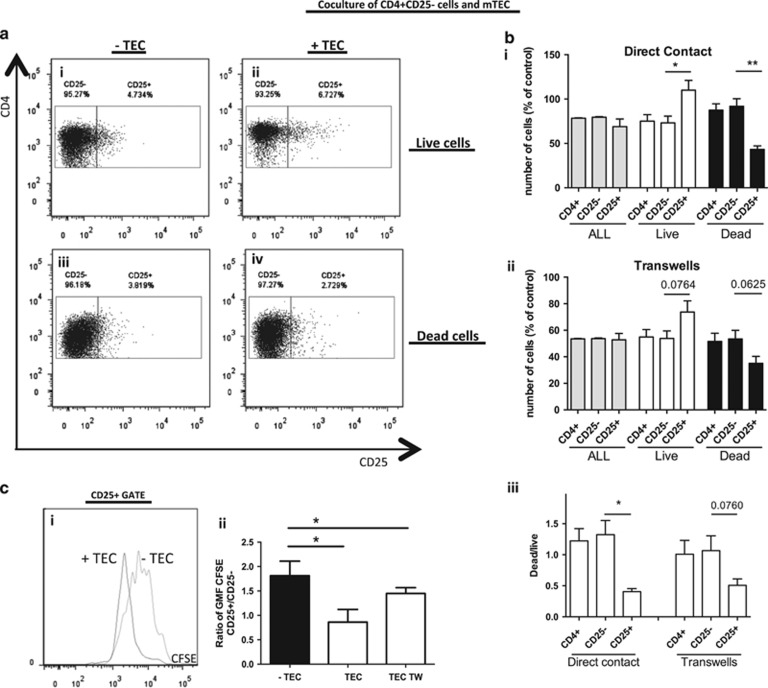
mTECs protect newly generated CD4+CD25+ cells from cell death and induce their preferential proliferation. CD4+CD25− T cells were cultured for 3 days in the presence or absence of mTECs. (**a**) Representative experiment by flow cytometry; the proportion of CD25− and CD25+ T-cell subsets was determined among the live and dead gates, as described in Supplementary Figures S2 (i–iv). (**b**) The absolute number of all (gray bars), live (white bars), or dead (black bars) CD4+, CD25−, and CD25+ cells was obtained after direct (i) or indirect (through TWs) (ii) culture with mTECs, and was calculated for each subset and normalized to the respective values without mTECs (*n*=4, mean±S.E.M.). Ratio of the number of dead cells and the number of live cells for all subsets (iii) (*n*=4, mean±S.E.M.). (**c**) The proliferation of the cells was evaluated using CFSE staining. Panel **c**i shows the effects of mTEC coculture on CD25+ cell proliferation. Results are summarized as a ratio of the GMF to CFSE in CD25+ cells over CD25− cells (ii) (*n*=4, mean±S.E.M.). Statistical analyses were conducted using one-way ANOVA followed by a Bonferroni *post-hoc* test for the figures in panel **b** and a non-parametric, paired *t*-test for panel **c**ii; *P<0.05, **P<0.005. *P* values between 0.1 and 0.05 are indicated

After 3 days of culture, both in the presence and in the absence of mTECs, a significant number of CD4+CD25+ cells were observed. The presence of these cells suggests that differentiation from CD25− to CD25+ cells occurs in both CD4 cell cultures and CD4/mTEC cocultures and that some CD25− Tconv cells purified at D0 were already engaged in the CD4+CD25+ cell differentiation process (Figure 5a). When we performed the analysis in a large gate including viable and dying cells (c.f. Supplementary Figure S2), we observed that the percentage of CD25+ cells was similar in the presence or absence of mTECs, suggesting that mTECs did not favor CD25+ cell differentiation (Figure 5a). However, the percentage of CD4+CD25+ cells in the live gate cells was higher in mTEC cocultures compared with CD4 cultures alone (mean value 7.6±1.4% in the presence of mTECs *versus* 5.2±1.0% in the control cultures; *P*<0.01; Figures 5ai and ii), whereas the percentage of CD25+ cells in the dead gate was lower in the mTEC coculture compared with the CD4 cultures alone (mean value 2.1±1.5% in the presence of mTECs *versus* 6.5±2.6% in the control cultures; *P*<0.01; Figures 5aiii and iv).

To further test whether mTECs affect the death of CD25+ and CD25− cells differentially, we analyzed the absolute number of cells in the different cell gates (Figure 5b). Coculture of CD4+CD25− cells with mTECs led to a decrease in the absolute number of CD4+ cells (22% decrease; Supplementary Figure S5b), which is in agreement with previous results obtained with total thymic cells.^26^ This decrease was not identical in the different subsets (Figure 5b). For cocultures indirect contact, there was no preferential effect on CD25− cells, whereas the number of live CD25+ cells strikingly increased and the number of dead CD25+ cells decreased (Figure 5bi). Similar results were observed in TW conditions (Figure 5bii). Thus, the ratio between dead and live cells is low in CD4+CD25+ cells (mean ratio=0.40) compared with CD4+CD25− cells (mean ratio=1.32), in both direct contact and TW conditions (Figure 5bii). The absolute numbers of live and dead cells among the relevant subpopulations (CD4+CD25+ and CD4+CD25− cells) are reported in Supplementary Figure S5 and confirm a lower number of dead CD25+ cells in the presence of mTECs or in TW conditions. These observations suggest that one of the effects of mTECs is to protect newly generated CD4+CD25+ T cells from cell death.

Next, we examined whether the protective effect on viable CD25+ cells could also be due to their preferential proliferation. We observed a shift of the CFSE peak to the left, in the CD25+ cells obtained after coculture (Figure 5ci). Data from four independent experiments confirmed that the CD25+ cells originating from CD25− cells were proliferating faster (a decrease in the CFSE GMF) than the CD25− cells (*P*<0.05; Figure 5cii) when cocultured with mTECs, suggesting that mTECs favor the proliferation of newly generated CD4+CD25+ cells.

Finally, using purified CD25+ cells in coculture with mTECs, we confirmed the protection of live CD25+ cells and the decrease in CD25+ dead cells (Supplementary Figures S6a and b). However, we did not observe an increased proliferation of purified CD25+ cells (Supplementary Figure S6c), suggesting that CD25− cells were necessary in this coculture system to favor CD4+CD25+ proliferation.

Our very detailed analyses of the different populations of CD4+ T cells clearly demonstrate that mTECs preferentially protect CD4+CD25+ Treg cells from cell death and can induce the proliferation of CD4+CD25+ T cells when they originate from the CD4+CD25− T-cell population, whereas no specific effect on CD4+CD25− cells was observed.

### mTEC protection of newly generated CD25+ cells involves the mitochondrial apoptotic pathway

As mTECs protect newly generated CD4+CD25+ cells from cell death, we investigated which pathway(s) was (were) responsible for that effect. We therefore investigated cellular changes associated with apoptosis using several specific cell death markers such as DilC1(5) (Mitoprobe), and CellEvent caspase assay kit that detects activated caspase-3 and -7.^27, 28^ In addition, we analyzed the expression of Bcl-2 that has an antiapoptotic function as it is involved in the intrinsic cell death signals that generally converge within the cell at the outer membrane of mitochondria.^29^

**Figure 6:**
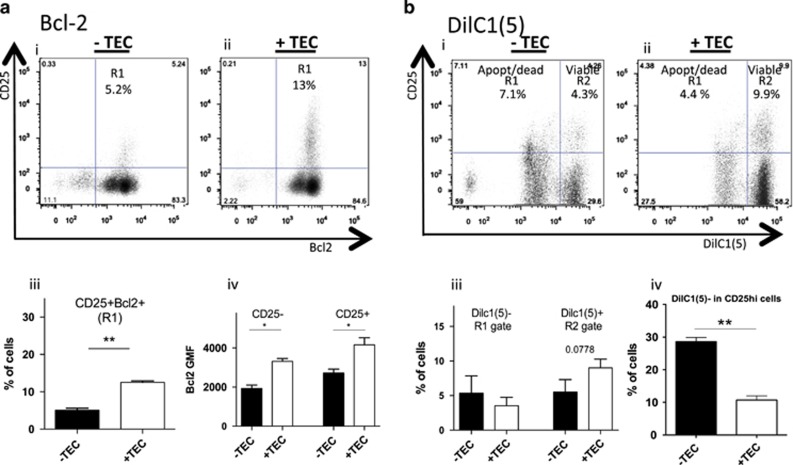
mTECs increase the expression of Bcl-2 and DilC1(5) in CD25+ cells. CD4+ cells were cocultured with or without mTECs for 3 days and different cell death pathways were investigated to identify the mechanism of protection of Treg cells by mTECs. (**a**) Bcl-2 expression was analyzed in permeabilized cells together with CD25 in absence (i) or presence (ii) of mTECs. The R1 gate represents Bcl-2+CD25+ cells. The mean values for R1 gate are shown in iii. The GMF of Bcl-2 is higher in CD25+ cells compared with CD25− cells (iv). (**b**) Analysis of DilC1(5) shows that CD25+DilC1(5)- cells (apoptotic/dead cells) (R1) decreased whereas the CD25+DilC1(5)+ cells (viable cells) (R2) increased in presence of mTECs (i and ii). The mean values for the R1 and R2 gates are shown in iii. In cells expressing high level of CD25 (CD25hi), the percentage of DilC1(5) is very much reduced in the presence of mTECs (iv). All experiments were repeated twice and are expressed as mean±S.E.M. Statistical analysis were conducted using a paired *t*-test; *P<0.05, **P<0.01

In the presence of mTECs, the percentage of CD25+Bcl-2+ cells increased significantly from 5±0.6 to 12.5±0.4 (Figures 6ai–iii). Bcl-2 GMF expression was higher in CD4+CD25+ cells compared with CD4+CD25− cells, and the expression increased in the presence of mTECs, suggesting that mTECs favor the expression of Bcl-2 in CD25+ cells (Figure 6aiv). As Bcl-2 is involved in the inhibition of the intrinsic cell death pathway that occurs through the mitochondria, we also analyzed the expression of DilC1(5) that measures the mitochondrial membrane potential (Figures 6bi–iv). Indeed during apoptosis there is a depolarization of the mitochondrial membrane, and as a result a reduced DilC1(5) fluorescence. In the presence of mTECs, the percentage of DilC1(5)+CD25+ viable cells (gate R2) increased from 5.5±1.3 (mean±S.E.M.) to 9±0.9%, whereas the percentage of DilC1(5)-CD25+ apoptotic/dead cells (gate R1) was reduced from 5.3±1.7 to 3.5±0.8 (Figure 6biii). The analysis in the cells expressing high level of CD25 (CD25hi) showed that the DilC1(5)− cells decreased strikingly when CD4+ cells were cocultured with mTECs (Figure 6biv). Finally, the involvement of caspase activation was investigated by two types of experiments: (1) The analysis of caspase activation in the CD4/mTEC coculture. Supplementary Figure S7a shows that the percentage of caspase-activated cells is much higher in CD25− cells compared with CD25+ cells. However, mTECs had no effect on these levels. (2) We asked whether inhibition of caspase could favor the Treg cell subset in the absence of mTECs. We used the Z-DEV-FMK inhibitor of caspase-3 and -7. Although it was able to limit the number of apoptotic cells, it did not promote Treg cell subset in the absence of mTECs (Supplementary Figure S7b), suggesting that blockage of apoptosis alone is not sufficient to preferentially promote Treg cells.

Altogether, these data show that CD4+CD25+ cells generated during the contact with mTECs are viable cells with high expression of Bcl-2 and high level of DilC1(5), suggesting that the intrinsic cell death pathway, also known as the mitochondrial apoptotic pathway, is involved in the protection of tTreg cells by mTECs.

### IL-2 overproduced by CD4+ cells in contact with mTECs is implicated in the mTEC effects

**Figure 7:**
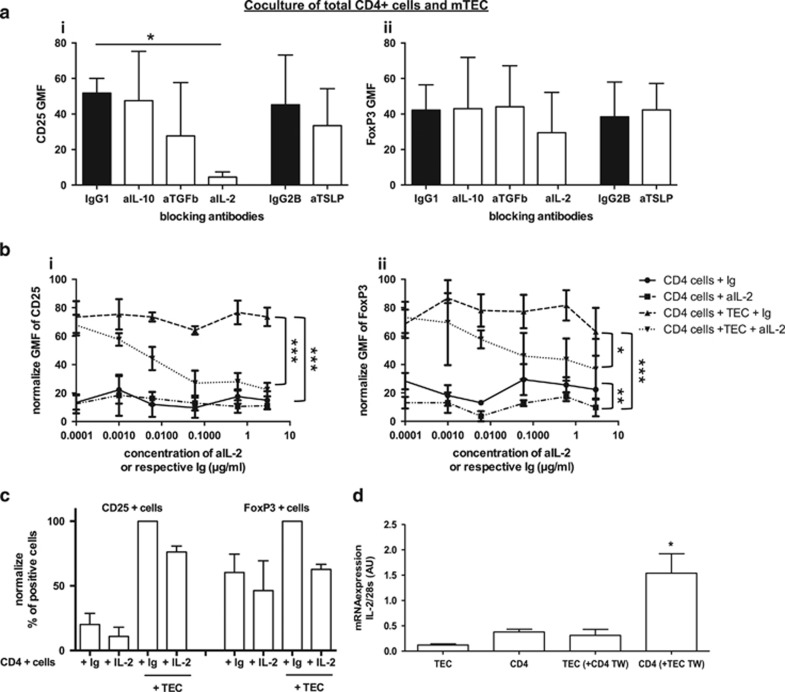
The protective effects of mTECs on Treg cell phenotype are mediated by IL-2. Purified CD4+ T cells were cultured alone or with mTECs for 3 days in the presence of different cytokines antagonists. (**a**) CD25 (i) and FoxP3 (ii) GMF expression in CD4 cells in different culture conditions were analyzed by flow cytometry (normalized data, *n*=3; mean±S.E.M.). The blocking antibodies against IL-10, TGF-*β*, and IL-2 were compared with their control isotype IgG1 and anti-TSLP was compared with its control isotype IgG2. (**b**) Purified CD4+ T cells were cultured alone or with mTECs for 3 days in the presence of increasing concentrations of IL-2 or its respective Ig. CD25 (**b**i) and FoxP3 (**b**ii) GMF were assessed by flow cytometry (*n*=3, mean±S.E.M.). To avoid the inter-experiment variability, data from each experiment were analyzed thanks to Graphpad ‘normalize' function, as described in Materials and methods. (**c**) The effects of IL-2 treatment (or its respective Ig) on the percentages of CD25 and FoxP3+ cells in the presence or absence of mTECs are reported in the graph. Statistical analyses were conducted using a two-way ANOVA test; *P<0.05, **P<0.005, ***P<0.001. (**d**) mRNA expression of IL-2 in mTECs or CD4+ cells, alone or in coculture (*n*=5, mean±S.E.M.). Statistical analyses were conducted using a non-parametric *t*-test; *P<0.05

We next elucidated which factors influence the generation of CD4+CD25+ cells in the presence of mTECs. Several cytokines, such as IL-2, IL-10, transforming growth factor (TGF)-*β*, and TSLP, mediate the Treg cell phenotype.^11^ We used blocking antibodies against these cytokines to identify which one could be implicated in our system (Figures 7ai and ii). Among them, only anti-IL-2 exhibited a significant inhibition of CD25/FoxP3 expression.

We then further analyzed the effects of increasing amounts of anti-IL-2 (0–5 *μ*g/ml) and found no effect on the CD25 GMF of CD4+ T cells when cultured alone, but observed a strong inhibition of the expression of CD25 in coculture with mTECs (Figure 7bi). Anti-IL-2 exhibited similar effects on the FoxP3 GMF (Figure 6bii), although these effects were incomplete. Interestingly, a slight but significant decrease in FoxP3 expression was observed when CD4+ T cells cultured alone were incubated with anti-IL-2 antibodies. Moreover, IL-2 reduced significantly the percentages of CD25 and FoxP3+ cells, although the effect was less striking than that observed for the GMF (Figure 7c). These results suggest that IL-2 may be involved in the effects of mTECs on CD4+CD25+ cells.

As mTECs did not produce significant levels of IL-2 (Figure 7d), we examined whether IL-2 production by CD4+ cells was regulated by mTECs. Figure 7d shows that CD4+ cells in the presence of mTECs produced higher levels of IL-2 compared with CD4+ cells alone. Since a higher proliferation of CD4+CD25+ cells was not observed in mTEC–CD4+CD25+ cocultures, but was observed in mTEC–CD4+CD25− cocultures, it is very likely that mTECs act on CD4+CD25− cells to induce higher levels of IL-2, which in turn promotes the generation of CD4+CD25+ cells.

### ICOSL is involved in the protection of CD4+CD25+ cells via the stimulation of IL-2 production by CD4+ cells

**Figure 8:**
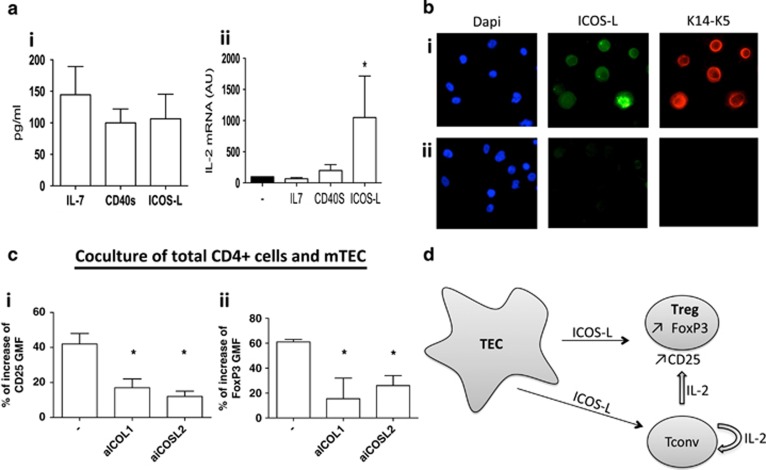
ICOSL is involved in the protection of CD25+ cells by mTECs. (**a**) (i) IL-7, CD40L, and ICOSL production in mTEC supernatants was measured by ELISA (*n*=5, mean±S.E.M.). (ii) IL-2 production after addition of IL-7, CD40S, or ICOSL to CD4+ cell cultures (*n*=2–7, mean±S.E.M.). Values are normalized to the condition without the addition of cytokines. A non-parametric, paired, one-tailed *t*-test was used for the statistical analysis. (**b**) ICOSL was stained in green by a specific antibody and mTECs (K14 and K5+) were stained in red on cytospins of cultured mTECs (i); the nuclei were stained with DAPI (magnification × 200). Staining with the respective immunoglobulins is shown in (ii). (**c**) Effects of two different concentrations of anti-ICOSL antibody (ICOSL1, 0.5 *μ*g/ml and ICOSL2, 1 *μ*g/ml) on the index of CD25 (i) and FoxP3 (ii) GMF expression of CD4 cells cocultured with mTECs (*n*=2, mean±S.E.M.). The index is calculated as the percentage of increase in the GMF of the cells in coculture normalized to cells cultured alone. The effects of ICOSL were analyzed via a one-tailed paired *t*-test, *P<0.05. (**d**) Proposed scenario for the action of mTECs on CD4+ cells. mTECs can produce ICOSL, which can directly interact with Treg cells or CD4 T cells. Coculture of CD4+ cells with mTECs leads to an enhanced production of IL-2 by Tconv cells, promoting Treg cell differentiation, proliferation, and survival

As cocultures with mTECs induce CD4+ T cells to overproduce IL-2, we determined which factors are responsible for this effect. ICOSL, sCD40, and IL-7 have been shown to stimulate Treg cell expansion.^21, 23, 30^ Quantification by enzyme-linked immunosorbent assay (ELISA) of mTEC supernatants showed that mTECs are indeed significant producers of IL-7, CD40s and ICOSL (Figure 8ai). When we tested whether these molecules could upregulate IL-2 production by CD4+ T cells, we observed that ICOSL was a significant inducer of IL-2 mRNA expression, whereas IL-7 or CD40s had no effect on IL-2 production (Figure 8aii). Moreover, the immunohistochemistry of cytospin of mTECs clearly shows that these cells (shown in red in Figure 8b; Keratin 14- and Keratin 5-stained cells) are producing ICOSL (in green).

We next demonstrated in our coculture system that inhibition of ICOSL with a blocking antibody decreased the CD25 and FoxP3 GMF (Figures 8ci and ii). In Figure 7, anti-IL-2 alone was not able to completely downregulate FoxP3 expression and the combination of anti-IL-2 and anti-ICOSL was no more effective than ICOSL alone. As anti-ICOSL has a more striking effect on FoxP3 than anti-IL-2, ICOSL likely directly affects FoxP3 expression.

These results suggest that the production of ICOSL by mTECs influences CD4+ Tconv to produce high levels of IL-2 in coculture, thus promoting the generation of Treg cells. The combined actions of IL-2 and ICOSL further facilitate this phenomenon (Figure 8d).

## Discussion

We summarize our work as follows: (1) mTECs protect Treg cell phenotype, (2) the effects are mTEC specific as they are not observed with other cell types, such as thymus myoid cells or CACO2 epithelial cells, (3) mTECs prevent newly generated Treg cells from cell death by protecting mitochondrial integrity and increasing Bcl-2, and promote their expansion, (4) these effects depend on soluble factors, as they do not require direct contact, (5) Tconv cells are essential mediators of this effect through IL-2 release, which is induced in the presence of mTECs, (6) mTECs produce ICOSL, which can stimulate IL-2 production by Tconv cells, and (7) blocking IL-2 and/or ICOSL leads to a major reduction in the effect on Treg cells. Altogether, our work shows that the fostering of thymus Treg cells involves intricate relationships between mTECs and Tconv CD4+ cells through soluble factors implicating IL-2 and ICOSL.

### mTECs have a major role in the development of Treg cells

The production of tTreg cells in the thymus is far from being well understood. In humans, very few studies have identified the nature of the stromal cells that influence the development of Treg cells. During development in mice, the appearance of Treg cells is delayed compared with that of Tconv cells.^10^ FoxP3+ cells are essentially localized in the thymus medulla, suggesting that this region provides a microenvironment to induce FoxP3 expression and Treg cell maturation.^31^ In mice, different types of stromal cells such as mTECs,^19, 32^ DCs,^33, 34^ and cTECs^12^ have been reported to foster Treg cell development. Thus, it seems that Treg cell differentiation does not require a dedicated thymus antigen-presenting cell.^33^ Similar to murine models, we show here that FoxP3+ human cells are located in the medulla. TSLP-conditioned mDCs can induce the differentiation of CD4+CD25− thymocytes into CD4+CD25+FoxP3+ Treg cells,^17^ but, in this case, TSLP was produced by mTECs from the Hassall's corpuscles, which are at a final stage of differentiation. Our culture system is highly enriched in mTECs and contains a very low number of DCs.^25^ Since the effects of DCs on inducing Treg cell induction depend on peptide–MHC class II interactions,^17^ whereas in our model the effects were essentially mediated by soluble factors, and since the addition of anti-TSLP did not counteract mTEC's effects, the results obtained in our system were certainly due to mTECs and not to contaminating DCs. Interestingly, other stromal cells were found to be able to induce the expansion of Treg cells. Mesenchymal stem cells recruit and regulate Treg cells.^35^ Similarly, human colonic myofibroblasts promote the expansion of Treg cells,^36^ but the addition of IL-2 is required. Although other cell types have been shown to foster Treg cells, the mechanism described here clearly implicates mTECs, which are the natural and physiological partners of CD4+ cells in the thymus, because of their proximity. A recent study describing a decrease in tTreg cells in mice with unpaired mTECs development supports our findings.^37^

### Evidence for a « ménage à trois »

The expansion of Treg cells by mTECs requires the presence of Tconv cells. During immune and autoimmune diseases, activated Tconv cells can boost Treg cell functionality. In a model of diabetes, when islet-specific Tconv cells were transferred alone, they induced diabetes, but when the same Tconv cells were co-transferred with islet-specific Treg cells, they induced disease protection by boosting Treg cell expansion and suppressive function.^38^ Tconv cells may act on Treg cell proliferation via production of IL-2, but their role in the generation of tTreg cells has never been described. In this work, we showed that coculture of purified tTreg cells with mTECs does not induce their expansion, but allows for the maintenance of their phenotype (CD25 and FoxP3), whereas a coculture of mTECs with Tconv cells induces a significant proliferation of Treg cells. These two findings could be explained by the absence of IL-2 in the system without Tconv cells.

### Molecular mechanisms involved in the generation of Treg cells

Many molecules appear to be important for the generation of tTreg cells. The CD40L/CD40 pathway has been implicated in tTreg cell differentiation, with a threefold decrease in tTreg cell frequency observed in mice deficient in either of those molecules.^30^ However, our data do not support a particular role of these molecules in our system. Although there are substantial data supporting the notion that TGF-*β* is important for the conversion of naive T cells into Treg cells, the function of TGF-*β* is clear in the periphery but controversial in the thymus.^11, 39^ Inhibition of TGF-*β* did not show any effect in our system. In addition, we performed high-scale analysis of the cytokines produced by mTECs via Raybiotech (Norcross, GA, USA) membranes (Supplementary Table S1), but most of the cytokines were below the detection levels. IL-6 and IL-8 were the main molecules detected. Inhibition of IL-6 was tested since IL-6 is known to alter Treg cell function,^40^ but we did not observe any change in CD25 expression in the presence of anti-IL-6 antibody (data not shown).

IL-2 is necessary for the expansion of Treg cells^10^ and mTECs do not produce IL-2. In our mTEC *in vitro* model, IL-2 had a major role as its neutralization significantly reduced the effects of mTECs on Treg cell phenotype, whereas anti-TGF-*β*, -IL-10, and -TSLP had no effect, suggesting no redundant factors able to increase CD25 levels. IL-2 is the primary molecule that enables the proliferation of Treg cells.^41^ In the absence of IL-2, the numbers of tTreg cells and pTreg cells are significantly reduced.^20, 42^ However, *in vitro* and *in vivo*, in addition to IL-2, IL-7 and IL-15 gamma chain-dependent cytokines transduce signals in tTreg cells.^21, 22, 43^ Indeed, in our system, IL-7 may be involved since a high level of IL-7 is produced under coculture conditions (data not shown) and is produced by mTECs. Furthermore, IL-7 can maintain FoxP3 expression.^41^ Even though this production was significantly increased at the protein level, the addition of IL-7 had no effect on IL-2 production by thymus CD4+ cells.

ICOSL produced by DCs is capable of stimulating the Treg cell phenotype.^23^ Analyses of the expression of ICOSL in our system showed that mTECs were capable of producing this factor. ICOSL is able to stimulate IL-2 production and blocking of ICOSL counteracts mTEC effects on T cells. Although other factors probably have additional effect in this system, the data obtained in this work highlight that IL-2 and ICOSL are the major factors of our system, since they can inhibit up to 70% and 65% of CD25 and FoxP3 expression, respectively, whereas TGF-*β*, IL-10, TSLP, and IL-6 (not shown) were not involved. This allows us to propose the following scenario (Figure 8d): mTECs produce ICOSL that interacts with its receptor on Tconv cells, which express high levels of ICOS after activation,^44^ and induces the production of IL-2. At the same time, the direct interaction of ICOSL with ICOS on Treg cells supports FoxP3 expression. IL-2 produced by Tconv cells promotes the proliferation of CD25+ cells. The feedback regulatory loop between Tconv and Treg cells may be critical to both limiting the development of autoimmune diseases^38^ and fostering the development of tTreg cells in the thymus.

Our work shows for the first time that human mTECs have a major role in Treg cell proliferation and protection and the expression of Treg cell markers. This mechanism, mediated by ICOSL, occurs via a direct effect on Treg cells and an indirect effect via stimulation of Tconv cells to produce IL-2.

## Materials and Methods

### Human sample collection

Normal thymus fragments were obtained from infants (aged 3 days to 4 years) and adults (13–30 years) undergoing cardiac surgery in hospitals located in Ile de France. These investigations were approved by the local Ethics Committee (‘Comité Consultatif de Protection des Personnes'), Ile de France VII (Kremlin Bicêtre, France). The relevant authorization numbers are ID RCB 2006-A00164-47 and 2010-A00250-39.

### Cell culture

All general cell culture products were obtained from Invitrogen (Cergy-Pontoise, France). The sera were obtained from Eurobio (Les Ulis, France).

All experiments performed in this paper use CD4-purified T cells, CD4+CD25− or CD4+CD25+ T cells, cultured either alone or with adherent cells for 3 days (optimal effects). Specific culture conditions for non-adherent or adherent cells, as well as cocultures, are described below.

### Adherent cell cultures

All the mTECs used in this work are primary mTECs obtained from fresh human thymus tissue and seeded onto cell culture flasks, as previously described.^45, 46^ Briefly, after removal of the thymic capsule, the thymic tissue was mechanically minced with scissors in Hanks buffer until 0.5 mm explants were obtained. After several washes in Hanks buffer, the explants were set down onto 75 cm^2^ flasks for 20 min to allow proper adhesion on the flask surface, and grown in RPMI 1640 GlutaMax (Invitrogen) medium supplemented with 20% of horse sera. Follow-up of the culture showed mTECs moving from the explants to the flask's surface around the explants. Extensive washings of the culture were done twice a week, to eliminate most of the thymocytes. mTECs (day 7–10 of culture) were trypsinized and seeded into 24-well plates for 4 h before the coculture experiments. The percentage of mTECs was estimated after 8 days of culture by immunofluorescence using an anti-keratin 14 and/or anti-keratin 5 antibody (c.f. the Immunohistochemistry section below) and was usually over 80%. The other cells were either contaminating fibroblasts or residual thymocytes.^25^ FACS-sorted mTECs could not be used in coculture experiments as they were not viable in culture.

The cell line culture of colorectal epithelial CACO2 cells was performed as described in the literature.^47^ A human stromal thymus myoid cell line was established in our laboratory as described in Wakkach *et al.*,^48^ and characterized.^26^ These cells are thymus muscle-like cells and were cultured in an RPMI 1640 GlutaMax I medium supplemented with 10% fetal calf serum.

### CD4 T cells

CD4 T cells were obtained from thymocytes isolated from fresh thymus tissue, as previously described in the literature.^49^ Total CD4+, CD4+CD25+, or CD4+CD25− cells were purified using magnetic separation according to the manufacturer's instructions (Dynabeads CD4+CD25+ Treg cell Separation Kit, Life Technologies, Saint Aubin, France, and CD4+CD25+ Treg cell Isolation Kit, Miltenyi, Paris, France), to which we added an anti-CD8 antibody (AbD Serotec, Düsseldorf, Germany) to eliminate CD8+ cells.

### Cocultures

Freshly purified thymocytes, such as CD4+, CD4+CD25+, or CD4+CD25− T cells, were seeded into 24-well plates in an RPMI 1640 Glutamax I medium supplemented with 10% fetal calf serum at 5 × 10^5^ cells per well, alone or with adherent cells. As different adherent cell types differ in their proliferation properties, cells were seeded in 24-well plates at 5 × 10^4^ cells per well for MITCs and 1 × 10^5^ cells per well for mTECs and CACO2 cells to reach subconfluence after coculture.

In some experiments (Figures 2, 3, 4, 5, 6), T cells were separated from adherent cells using cell culture insert TWs (1 *μ*m pore size, Becton Dickinson, Le-Pont-de-Claix, France), to prevent cell contact but to allow diffusion of soluble mediators.

In some experiments (Figures 6 and 7), blocking antibodies were used at the following concentrations: anti-IL-10 at 5 *μ*g/ml, anti-IL-2 between 0 and 5 *μ*g/ml, anti-TSLP at 0.1 *μ*g/ml, anti-TGF-*β* at 5 *μ*g/ml, and anti-ICOSL between 0.5 and 1 *μ*g/ml. All antibodies were from R&D Systems, Lille, France. Control isotypes IgG1 and IgG2B (R&D Systems) were used at the same concentrations as their corresponding antibody.

### Suppressive assay

The suppressive activity of CD4+CD25+ cells following 3 days of culture with mTECs was evaluated by tritiated thymidine incorporation, as previously described in the literature.^45^ The suppressive capacity of Treg cells was normalized as the percentage of proliferative response of Tconv cells alone (*n*=3, mean±S.E.M.).

### Flow cytometry

To analyze Treg cell phenotype, purified CD4+ T cells were stained with anti-CD4 (mouse APC-conjugated anti-human CD4, DAKO, Trappes, France) and anti-CD25 (mouse phycoerythrin-Cy7-conjugated anti-human CD25, Becton Dickinson) antibodies for 30 min at 4 °C before permeabilization with the FoxP3 permeabilization kit (eBioscience, Paris, France) and labeling with anti-FoxP3 (rat phycoerythrin-conjugated anti-human FoxP3, eBioscience) according to the manufacturer's instructions.

The proliferation of CD4+ cells was evaluated using CFSE (Sigma-Aldrich, Lyon, France) labeling according to the manufacturer's instructions.

To analyze the cell death pathway involved in our system, we used several assays to evaluate the changes in the apoptotic stage of Treg cells when the CD4+ cells were cocultured or not with mTECs. DilC1(5) is a mitoprobe that shows mitochondrial membrane potential changes during early apoptosis. We used the DilC1(5) Assay kit for flow cytometry (M34151) from Molecular Probes (Invitrogen Detection Technologies, Life Technologies), according to the recommendation of the manufacturer. The inhibition of caspase-3 and -7 was studied by using the Z-DEV-FMK inhibitor (ALX-260-141-R100) from Enzo and was used at a final dilution of 10 *μ*M on whole CD4+ cells for 2 h. The cells were then washed and cultured for 3 days before the analysis of the Treg cell phenotype. Caspase-3 and -7 (Casp3/Casp7) activation was analyzed by using the CellEvent Caspase-3/7 Green Flow Cytometry Assay Kit from Molecular Probes (Invitrogen Detection Technologies) and was used according to the recommendation of the manufacturer. The expression of Bcl-2 was analyzed using a monoclonal mouse anti-Bcl-2 antibody conjugated to FITC (F7053, DAKO) after permeabilization of the cells as recommended by the manufacturer. All apoptotic markers were used together with anti-CD4 and anti-CD25 antibodies as described above.

Acquisition was performed on Becton Dickinson cytometers (FACS Calibur—acquisition software Cell Quest, FORTESSA II—acquisition software FACS Diva, FACS Verse—acquisition software FACS Suite) and subsequent analysis was performed using FlowJo software (Treestar, Olten, Switzerland) or the FACS Suite (Becton Dickison, Le-Pont-de-Claix, France).

All cytometry experiments except those with the results shown in Figure 5 and Figure 6 were performed as follows: after gating on living cells (according to their forward- and side-scattered light,^50^ c.f. (Supplementary Figure S2), the GMF was evaluated for the FoxP3 and CD25+ gates of CD4+ cells. In Figure 5, after gating on CD4+ cells, CD25− and CD25+ subsets were determined in live and dead cell gates (Supplementary Figure S2). In Figure 6, the analysis was done in a large gate including the live and dead/apoptotic cells, and then in gating in CD4+ cells.

### Immunohistochemistry

In Figure 1, fluorescent staining was performed on 7 *μ*m of acetone-fixed, frozen thymic sections. The following primary antibodies were used: rabbit polyclonal anti-human keratin (Biogenesis LTD, Poole, England) and mouse phycoerythrin-conjugated anti-human FoxP3 (eBioscience). Polyclonal anti-keratin antibody was detected by incubation with the following secondary antibodies: goat Alexafluor 488-conjugated anti-rabbit (Life Technologies). After 20 min of fixation with acetone, the primary antibodies were incubated for 1 h at room temperature followed by three phosphate buffered saline (PBS) washes before incubation with the secondary antibody (1 h at room temperature) and mounting. Slides were mounted in Faramount fluorescent mounting media (DAKO). Images were acquired with a Zeiss (Marly Le Roi, France) Axio Observer Z1 Inverted microscope with a × 10 eyepiece objective and a × 10 or × 20 objectives, with a Zeiss AxioCam MRm camera. The acquisition software was Axiovision (Zeiss).

In Figure 7, fluorescent staining was performed on acetone-fixed (10 min), cytospin mTECs after 8 days of culture. The following primary antibodies were detected: rabbit polyclonal anti-human keratin 14 and anti-human keratin 5 (Eurogentec, Angers, France), and mouse monoclonal anti-human ICOSL (R&D Systems). The respective secondary antibodies were chicken Alexafluor 594-conjugated anti-rabbit (Life Technologies), and goat Alexafluor 488-conjugated anti-mouse (Life Technologies). The primary antibodies were incubated for 2 h at room temperature, followed by three PBS washes before incubation with the secondary antibody (1.5 h at room temperature), and again followed by three PBS washes before incubation with DAPI staining, three final PBS washes, and mounting. All fluorescent staining was compared with staining with the respective primary antibodies immunoglobulins (IgG2B anti-mouse, IgG anti-rabbit, DAKO). Slides were mounted in fluorescent mounting media (DAKO). Images were acquired with a Zeiss Axio Observer A1 Microscope with a × 10 eyepiece objective and a × 20 objective, with a Zeiss AxioCam MRm camera. The acquisition software was Axiovision.

### ELISA

ELISA analyses were performed following the manufacturer's instructions for the different cytokines tested (IL-7, Peprotech, Neuilly sur Seine, France; CD40s, R&D Systems; ICOSL, Antibodies-online GmbH, Paris, France) on 100 *μ*l of cell culture supernatant. Measurements were performed on a MRX Revelation microplate reader from Dynex (Thermolab System, Chantilly, VA, USA).

### qPCR analyses

For RNA extraction and further qPCR analysis experiments (Figures 7a and b), cytokines were added for 24 h to CD4+ cultures at the following concentrations: IL-7 (0.75 ng/ml, Peprotech), CD40S (25 ng/ml, R&D Systems), and ICOSL (0.125 *μ*g/ml, Antibodies-online GmbH).

Extraction of total RNA from CD4+ T cells and synthesis of cDNA was performed as previously described in the literature.^49, 51^ We performed qPCR using reagents and a LightCycler 480 system provided by Roche SAS (Boulogne-Billancourt, France) with FOXP3 primers,^49^ CD25 primers (F: 5′-ATCAGTGCGTCCAGGGATAC-3′ R: 5′-GACGAGGCAGGAAGTCTCAC-3′), and IL-2 primers (F: 5′-ACCTCAACTCCTGCCACAAT-3′ R: 5′-GCCTTCTTGGGCATGTAAAA-3′). Acquisition was performed with the Roche LightCycler Software version 1.5. The results are normalized to 28S.^49, 52^

### Statistical analyses

Differences between groups were evaluated by two-way ANOVA and parametric or non-parametric *t*-tests for paired or unpaired data (InStat, GraphPad Software, San Diego, CA, USA), with the significance level set to *P*<0.05.

In some experiments, the ‘normalize' function of GraphPad was used to limit the inter-experiment variability owing to the use of different FACS machines. This function recalculates the values within a data set considering the highest value to be 100% and the lowest value to be 0%.

## Abbreviations

Bcl-2: B-cell lymphoma 2
cTECs: cortical TECs
DAPI: 4',6-diamidino-2-phenylindole
DCs: dendritic cells
ELISA: enzyme-linked immunosorbent assay
FoxP3: forkhead box P3
GMF: geometric mean fluorescence
ICOSL: inducible T-cell costimulator ligand
MITCs: myoid immortalized thymic cells
mTECs: medullary thymus epithelial cells
pTreg: peripheral Treg
qPCR: quantitative real-time PCR
S-TEC: supernatants from mTEC cultures
TGF: transforming growth factor
TSLP: thymus stromal lymphopoietin
tTreg: thymic T regulatory
TWs: transwells
