# Supplementary material for: Human thymus medullary epithelial cells promote regulatory T-cell generation by stimulating interleukin-2 production via ICOS ligand
Source: Cell Death Dis. 2014 Sep 11;5(9):e1420–. doi: 10.1038/cddis.2014.377 (PMC4540205; doi:10.1038/cddis.2014.377)
Supplement: Supplementary Information [file cddis2014377x1.pdf]

Supplemental data

a

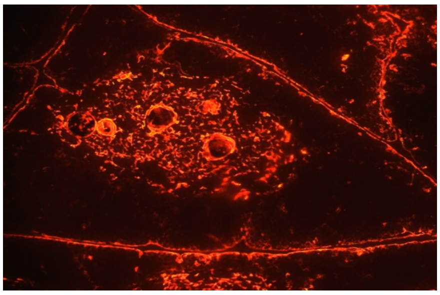

b

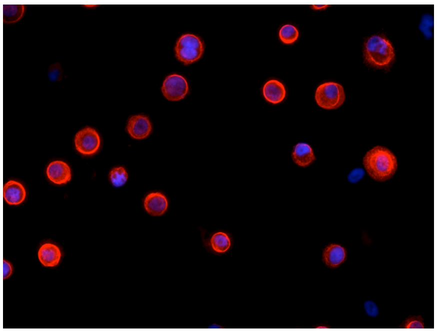

c

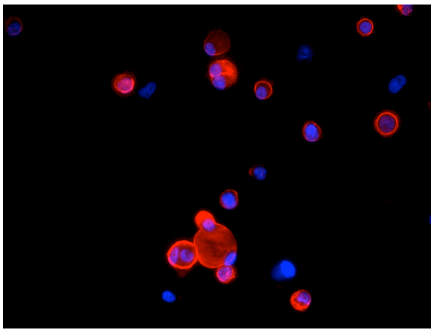

Figure S1. Expression of keratin 14 and 5 in the human thymus and in the cultured mTEC. (a) Human thymic section stained with anti-keratin 14 antibody shows a clear delineation of the medullary area of the thymus. Medullary epithelial cells and the subcapsular epithelial cells are stained with the anti-keratin 14 antibody while the cortical epithelial cells are negative. (b) and (c) Cytospin of medullary thymic epithelial cell cultures; more than 85% of the cells react with a mixture of anti-keratin 5 and anti-keratin 14 antibodies.

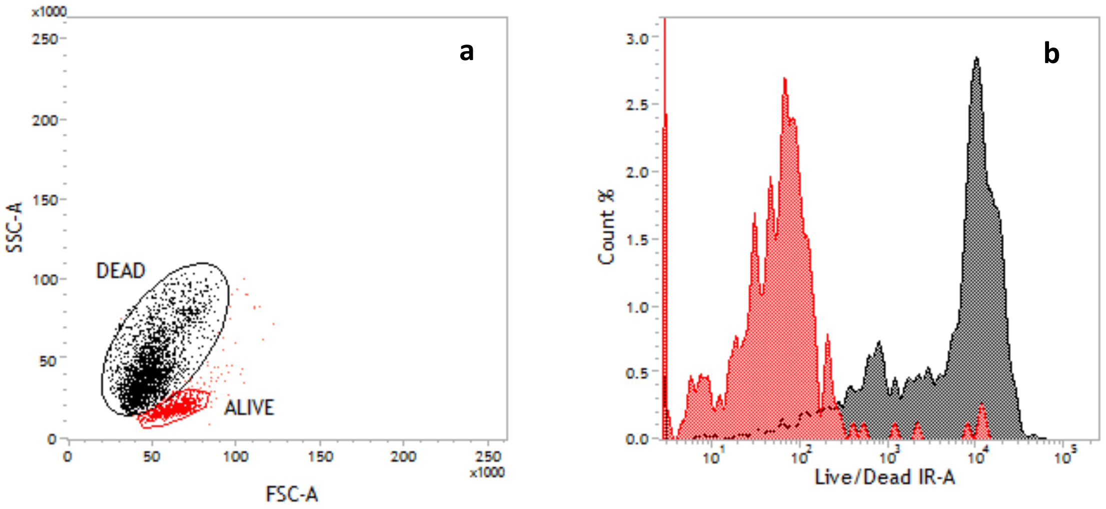

Figure S2. Identification of cell gates according to cell viability. Cell viability was assessed with a live/dead Infra Red staining from Life Technologies (Saint Aubin, France). The FSC/SSC position of the cells (a) matches the marker of viability. In panel (b), the cells exhibiting the marker were dying or dead cells.

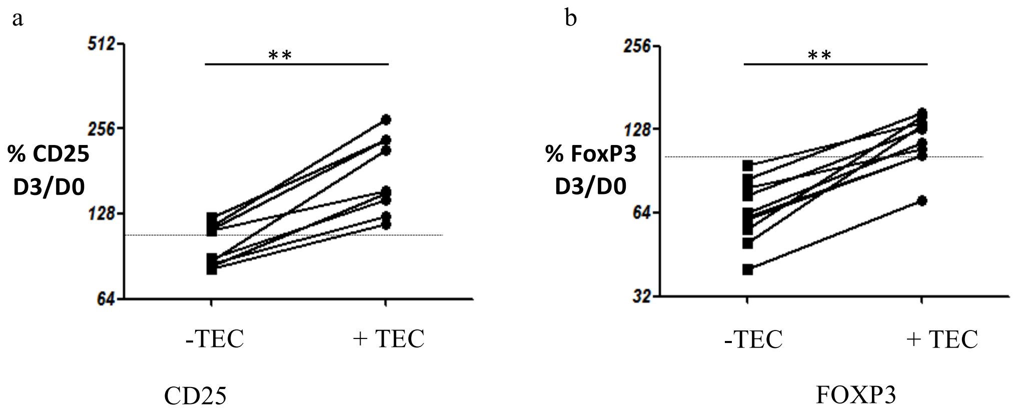

Figure S3. Percentage of CD25 and FoxP3 positive cells in the CD4+ cell cultures. The percentages of CD25 (a, $\log_2$ axis; n=9) and FoxP3 (b, $\log_2$ axis; n=10) positive cells after 3 days of coculture were normalized as a percentage of the D0 value, represented on the graph by the dotted line. Statistical analysis were conducted using a non-parametric, paired t-test.

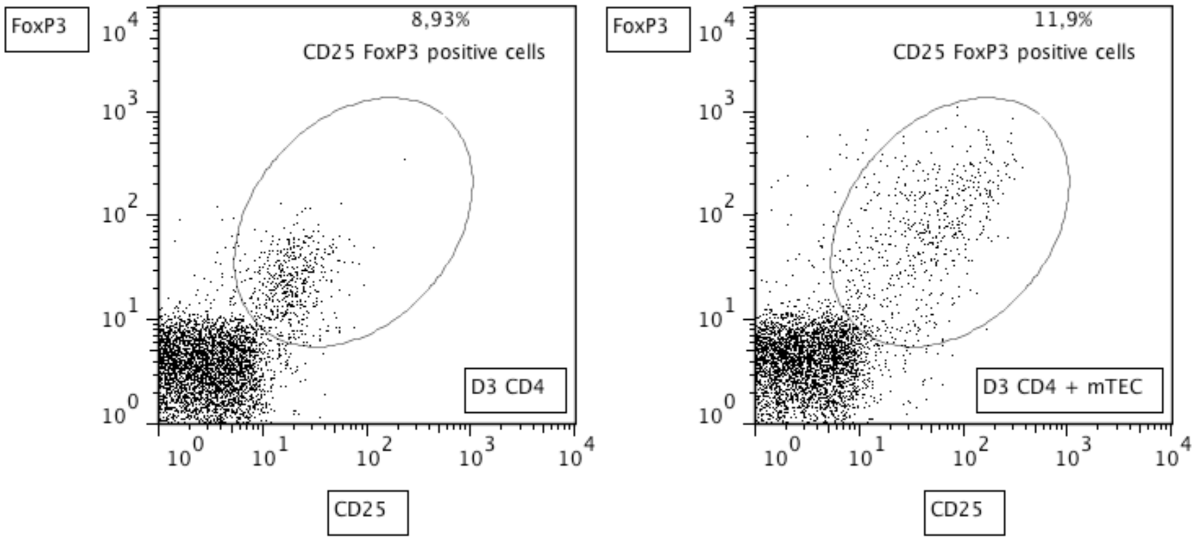

Figure S4. Expression of CD25 and FoxP3 in CD4+ cells. CD4+ cells were cultured alone or with mTECs for 3 days. After culture, double positive CD25 and FoxP3 cells were more numerous in CD4+ mTECs coculture. Few cells were either CD25 or FoxP3 single positive cells, but the majority of the cells were double CD25 FoxP3 positive cells.

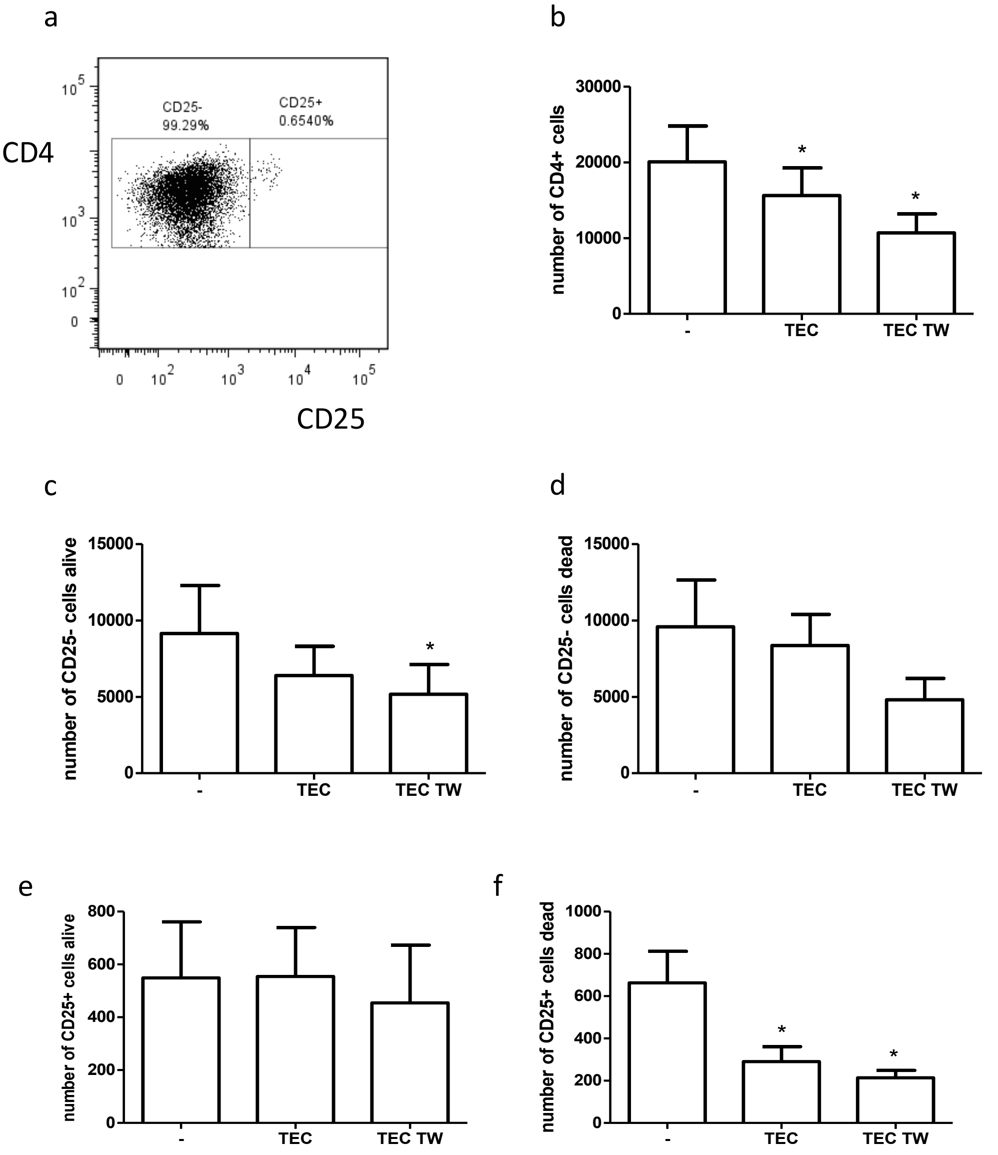

Figure S5. Flow cytometry analysis of T cells before and after culture. (a) Purity of CD4+CD25- cells before coculture. (b) Number of CD4+ cells after coculture. (c–d) Number of CD25- living and dead cells after coculture. (e–f) Number of CD25+ living and dead cells after coculture. For all panels, *n*=4, mean ± sem. Statistical analysis were conducted using a paired *t*-test.

a

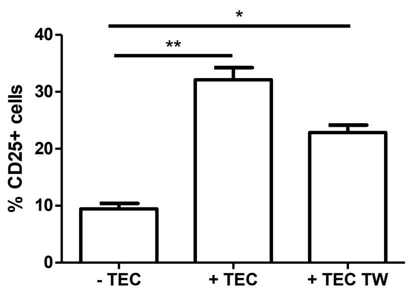

b

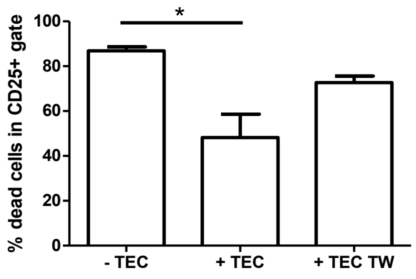

c

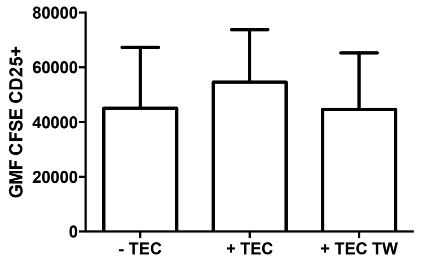

Figure S6. mTECs preserved the CD4+CD25+ phenotype in cocultures of purified Tregs. (a) The percentage of CD25+ cells was maintained in Treg cocultures with mTECs. (b) Treg cells were protected from cell death by mTEC coculture. (c) Coculture with mTECs did not promote proliferation of Treg cells as the GMF of CFSE in CD25+ cells alone did not differ from that in cultures of Tregs alone (n=3; mean ± sem). The data were analyzed by a one-way ANOVA test, followed by a post-hoc Bonferroni test.

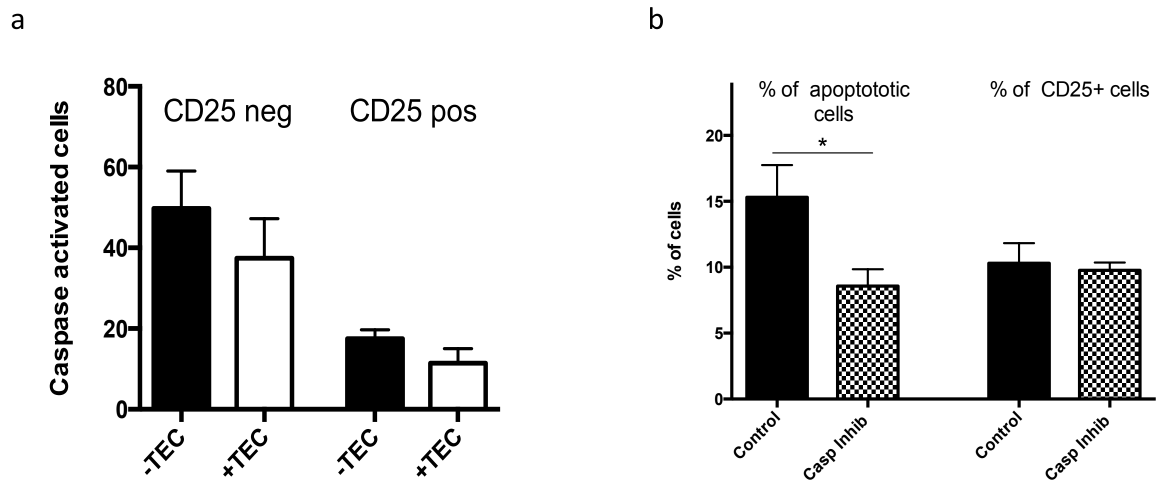

Figure S7. Role of caspases in the protection of Treg phenotype

(a) In CD4/mTEC coculture, the percentage of cells presenting activation of Casp3/Casp7 was evaluated by the Cell event Caspase 3/7 Green Flow cytometry Assay kit. The percentage of caspase-activated cells was higher in CD25- compared to CD25+ cells, but the presence of mTEC did not significantly change the percentage of caspase-activated cells (n=2). (b) The inhibitors of caspase-3 and -7 (Z-DEV-FMK (Enzo, ref ALX-260-141-R100) was used at a final dilution of 10 μM on whole CD4+ cells for 2 hours, then washed and cultured for 3 days to investigate whether they could protect the CD4+CD25+ phenotype. The percentage of apoptotic cells was decreased in presence of the inhibitor. The percentage of CD25+ cells was unchanged in presence or absence of the caspase inhibitor (n=3, mean ± sem).

Cytokine	Level (AU)
IFN-gamma	46
IL-1 alpha	52
IL-1 beta	42
IL-16	undetectable
IL10	57
IL12-p40/p70	undetectable
IL13	undetectable
IL15	undetectable
IL2	undetectable
IL3	undetectable
IL4	undetectable
IL5	undetectable
IL6	147
IL7	58
IL8	143
TGF beta 3	undetectable
TGF beta1	44
TGF beta2	75
TNF- beta	52
TNF-alpha	76

Table S1. Cytokine production by mTECs in culture.

The levels of cytokines were evaluated by a semi-quantitative Raybiotech membrane (Human cytokine array, C-Series, Raybiotech) in 3 different mTECs cultures. The membranes were scanned and the density of the spots was evaluated. The intensity levels are given as arbitrary units (AU). Values lower than 30 were considered as undetectable. Many cytokines were below the background, some were slightly positive (light orange); The 2 major cytokines were IL-8 and IL-6 (deep orange).
